# Birth of the blues: emotional sound processing in infants exposed to prenatal maternal depression

**DOI:** 10.1017/S0033291720002688

**Published:** 2022-08

**Authors:** Michael C. Craig, Vaheshta Sethna, Maria Gudbrandsen, Carmine M. Pariante, Trudi Seneviratne, Vladimira Stoencheva, Arjun Sethi, Marco Catani, Mick Brammer, Declan G. M. Murphy, Eileen Daly

**Affiliations:** 1 Department of Forensic & Neurodevelopmental Sciences, Sackler Institute for Translational Neurodevelopment, Institute of Psychiatry, Psychology & Neuroscience, King's College London, London, UK; 2 Natbrainlab, Department of Forensic & Neurodevelopmental Sciences, Institute of Psychiatry, Psychology & Neuroscience, King's College London, London, UK; 3 National Female Hormone Clinic, Maudsley Hospital, SLAM NHS Foundation Trust, London, UK; 4 Stress, Psychiatry and Immunology & Perinatal Psychiatry Laboratory, Institute of Psychiatry, Psychology & Neuroscience, King's College London, London, UK; 5 Perinatal Services, Maudsley Hospital, SLAM NHS Foundation Trust, London, UK

**Keywords:** Emotional sounds, fMRI, infants, prenatal maternal depression

## Abstract

**Background:**

Offspring exposed to prenatal maternal depression (PMD) are vulnerable to depression across their lifespan. The underlying cause(s) for this elevated intergenerational risk is most likely complex. However, depression is underpinned by a dysfunctional frontal-limbic network, associated with core information processing biases (e.g. attending more to sad stimuli). Aberrations in this network might mediate transmission of this vulnerability in infants exposed to PMD. In this study, we aimed to explore the association between foetal exposure to PMD and frontal-limbic network function in infancy, hypothesising that, in response to emotional sounds, infants exposed to PMD would exhibit atypical activity in these regions, relative to those not exposed to PMD.

**Method:**

We employed a novel functional magnetic resonance imaging sequence to compare brain function, whilst listening to emotional sounds, in 78 full-term infants (3–6 months of age) born to mothers with and without a diagnosis of PMD.

**Results:**

After exclusion of 19 datasets due to infants waking up, or moving excessively, we report between-group brain activity differences, between 29 infants exposed to PMD and 29 infants not exposed to PMD, occurring in temporal, striatal, amygdala/parahippocampal and frontal regions (*p* < 0.005). The offspring exposed to PMD exhibited a relative increase in activation to sad sounds and reduced (or unchanged) activation to happy sounds in frontal-limbic clusters.

**Conclusions:**

Findings of a differential response to positive and negative valanced sounds by 3–6 months of age may have significant implications for our understanding of neural mechanisms that underpin the increased risk for later-life depression in this population.

## Background

Approximately 15% of women suffer from a major depressive episode during pregnancy (Vesga-Lopez et al., 2008). Further studies have found that offspring exposed to prenatal maternal depression (PMD) are vulnerable to depression across their lifespan (Pawlby, Hay, Sharp, Waters, & O'Keane, 2009) – independent of the effects of postnatal depression (Pearson et al., 2013). The underlying cause(s) for this elevated intergenerational risk is most likely complex [see Lewis (2014) for review]. Potential mechanisms mediating this risk include transmission of brain processing aberrations, underpinned by similar dysfunctional neural network(s) to those reported in later-life depression (Zeng et al., 2012). For example, in response to negative/sad stimuli, these typically include hyper-activity of amygdala and para-limbic structures (Beesdo et al., 2009; Fu et al., 2004; Surguladze et al., 2005), and inhibition of prefrontal cortical activity (Fu et al., 2004; Siegle, Thompson, Carter, Steinhauer, & Thase, 2007; Zhong, Pu, & Yao, 2016). Also, in response to positive/happy stimuli, non-depressed individuals typically exhibit increased activity in bilateral fusiform gyri and right putamen, compared to depressed individuals (Surguladze et al., 2005). Similarly, differences in brain activity are found in individuals at high risk of depression even in the absence of personal history of depressive episodes (Chan, Harmer, Goodwin, & Norbury, 2008; Mannie et al., 2008). These findings are consistent with negative information processing biases (e.g. attending more to sad stimuli), which are a core feature of people that develop clinical depression (Gotlib, Krasnoperova, Yue, & Joormann, 2004). Similar attention biases have been reported in children born to depressed mothers (Gibb, Benas, Grassia, & McGeary, 2009; Joormann, Talbot, & Gotlib, 2007; Kujawa et al., 2011). Further, a recent imaging study of 6–9 years old exposed to elevated PMD ‘symptoms’ reported increased amygdala activation, to negative emotional faces (van der Knaap et al., 2017). The latter study was an important next step. However, it was limited by the absence of clinically depressed mothers and recruitment of older children, where later environmental influences (e.g. social environment and ongoing maternal depression) are more difficult to disentangle from prenatal effects (Pawlby et al., 2009). To limit the putative effect of such influences, we and others have pioneered techniques that allow imaging of infants shortly after birth. This has led to recent findings of atypical amygdala–prefrontal connectivity in infants exposed to PMD (Cha et al., 2017) and neonates exposed to prenatal maternal stress (Lautarescu et al., 2020) – supporting the hypothesis that aberrations in this network may mediate transmission of risk to later-life depression in infants exposed to PMD. We have further extended this approach and developed a novel fMRI sequence that permits analysis of the infant neural response to auditory emotional stimuli (Blasi et al., 2011, 2015). This approach has permitted us to obtain task-related fMRI data, whilst minimising motion artefacts associated with infants during an awake state.

Therefore, in this prospective study, we used task-based fMRI to analyse brain activation in 3–6 months old infants born to mothers with, and without, a clinical diagnosis of prenatal major depressive disorder (MDD) confirmed during pregnancy. We hypothesised that, in response to sad sounds, infants born to prenatally depressed mothers would exhibit hyper-activity of amygdala and para-limbic structures with reduced prefrontal cortical activity; and in response to happy sounds, hypo-activation in bilateral fusiform gyri and right putamen, relative to offspring of mothers without PMD.

## Methods

### Participants

Overall, 78 full-term infants (37–42 weeks' gestation) aged 3–6 months, with normal birth weight (>2500 g), were recruited after mothers were approached, predominantly during their second and third trimester, from antenatal clinics and perinatal psychiatric services within the same community in South East London between 2011 and 2014. They included, respectively, 41 infants born to mothers without PMD and 37 infants exposed to mothers diagnosed with PMD. All mothers without PMD had been screened as being ‘low-risk’ by midwives. This included the absence of a past history of major mental health problems and/or clinically significant depressive symptoms at the material time. The mothers with PMD were subsequently assessed by a psychologist using the Structured Clinical Interview for DSM-IV Axis I Disorders (SCID-I) (First, Spitzer, Gibbon, & Williams, 1997) concerning their prenatal status at 32 weeks and depressive symptoms were further quantified in all mothers using the Beck Depression Inventory (BDI) (Beck & Steer, 1993).

Exclusion criteria included mothers without a working knowledge of the English language, past MDD or other current or past major mental disorders (e.g. bipolar disorder or psychosis), reported substance use disorders, psychotropic medication other than antidepressants (i.e. in the PMD group), significant antenatal or obstetric complications potentially altering infant development (e.g. gestational diabetes, placental anomalies), hearing deficits, or any contraindications for MRI scanning.

The authors assert that all procedures contributing to this work comply with the ethical standards of the relevant national and institutional committees on human experimentation and with the Helsinki Declaration of 1975, as revised in 2008. All procedures involving human subjects/patients were approved by the UK National Research Ethics Committee (REC08/H0718/76,06/MRE02/73,12/LO/2017). Written informed consent was obtained from all women.

### Experimental design

#### Testing procedure

Scanning sessions were co-ordinated around the time of infants' natural sleep. To reduce movement during imaging, infants were swaddled in a cotton sheet within a MedVac Vacuum Immobilization Bag (DFI Medical Solutions). Scanner noise was minimised by lining the scanner bore with sound attenuating foam. Also, infants' ears were covered by Natus MiniMuffs Noise Attenuators, followed by MR-compatible piezoelectric headphones (http://www.mr-confon.de/en), which also enabled the presentation of the auditory stimuli.

#### Stimuli

We presented infants with our standard battery of stimuli, within a paradigm that we have previously validated in this age group (Blasi et al., 2011, 2015). These included three categories of adult male and female ‘non-speech vocalisations’: *emotionally neutral* (coughing, sneezing or yawning), *positive* (laughter) and *negative* (crying) sounds (The Montreal Affective Voices). Infants were also presented with ‘non-vocal’ *environmental* (running water and toy) sounds (The Voice Neurocognition Laboratory). Auditory stimuli were presented using a block design, to optimise statistical power, comprising 32 blocks (eight per stimulus category), separated by 9 s rest, lasting 16 min in total. Each 21 s block contained 7–11 different sounds separated by 0.47–0.75 s.

#### FMRI data acquisition

MRI data were acquired using a GE 1.5 Tesla Twinspeed MRI scanner (General Electric, Milwaukee, WI, USA). The body coil was used for RF transmission and an eight-channel head coil for RF reception procedure (Simmons, Moore, & Williams, 1999). Gradient rise times were limited in order to reduce the noise of the pulse sequences to approximately 70 dB. Daily quality assurance was carried out to ensure high signal to ghost ratio, high signal to noise ratio and excellent temporal stability using an automated quality control procedure (Simmons et al., 1999).

#### FMRI sequence

The 320 T2* weighted gradient echo planar multi-slice datasets depicting BOLD (Blood Oxygenation Level Dependent) contrast were acquired in each of 24 non-contiguous near-axial planes (4.0 mm thick with 2.0 mm spacing, 3.5 × 3.5 mm in-plane resolution) parallel to the Anterior Commissure-Posterior Commissure (AC-PC) line (TE 57 ms, TR 3000 ms, flip angle 90°, number of signal averages = 1, 16:04 min).

#### Structural sequence

A T2 weighted fast spin echo dataset was acquired (256 × 168 rectangular matrix, 2 mm slice thickness, 0 mm slice gap, field of view = 18 cm, TR = 4500, TE = 115 ms, echo train length = 17).

### Data analysis

#### Descriptive statistics

Group differences between mothers and infants from both groups were calculated using IBM SPSS Statistics 22 (Table 1).

**Table 1.** Maternal and infant demographic and clinical characteristics

	Antenatally depressed (*n* = 29)	Non-depressed (*n* = 29)	Group differences Statistic (*p* value)
Infant demographics *mean* (s.d.)			
Age at MRI (days)	148 (40)	151 (39)	*t* = −0.24, *p* = 0.81
Gestational age at birth (weeks)	39.7 (2.1)	39.6 (1.9)	*t* = 0.12, *p* = 0.90
Birth weight (grams)	3416 (527)	3315 (703)	*t* = 0.61, *p* = 0.55
Weight at MRI (grams)	7310 (1175)	7223 (1397)	*t* = 0.26, *p* = 0.80
Gender; *n* (%) male	17 (59)	17 (59)	χ^2^ = 0.00, *p* = 1.00
Maternal demographics *mean* (s.d.)			
Age (years)	32.5 (4.2)	33.2 (4.6)	*t* = −0.57, *p* = 0.57
Educational level *n* (%)			
GCSE and A-Levels	7 (24.1)	2 (6.9)	χ^2^ = 3.56, *p* = 0.19
Vocational college	3 (10.3)	3 (10.3)	
Higher education	19 (65.5)	24 (82.8)	
On antidepressant treatment *n* (%)			
Pregnancy	13 (44.8)	0 (0)	χ^2^ = 16.76, *p* < 0.001
Postnatal	15 (51.7)	1 (3.4)	χ^2^ = 16.92, *p* < 0.001
BDI score* mean (s.d.)			
Prenatal BDI (32 weeks)	25.6 (12.7)	7.5 (4.0)	*t* = 6.90, *p* < 0.001
Breastfeeding *n* (%)			
Yes	21 (72)	23 (79)	χ^2^ = 1.40, *p* = 0.238
Marital status *n* (%)			
Single (no partner)	4 (14)	1 (4)	χ^2^ = 4.40, *p* = 0.222
Single (with partner)	4 (14)	1 (4)	
Cohabiting	6 (21)	7 (24)	
Married	15 (52)	20 (69)	

s.d., standard deviation; GCSE, General Certificate of Secondary Education; A-Levels, General Certificate of Education Advanced Level; Higher Education, undergraduate and postgraduate degree; BDI, Beck Depression Inventory *(0–13: minimal depression; 20–28: moderate depression).

#### FMRI data analysis

MRI data were analysed using XBAM (http://www.kcl.ac.uk/ioppn/depts/neuroimaging/research/imaginganalysis/software/XBAM.aspx) using a data-driven approach. Minimization of motion-related artefacts and removal of linear trends was carried out with a rigid-body transform to account for translation and rotation using a spin history correction (Bullmore et al., 1999). After registration, the 3D images were realigned at each time point by finding the combination of rotations (around the three axes) and translations (in three dimensions) that maximized the correlation with an image obtained by averaging the intensity at each voxel over the whole experiment. Then, data were smoothed using a Gaussian filter (7.2 mm isotropic FWHM). Each component of the experimental design was convolved with two *γ* variate functions (peak responses at 4 and 8 s, respectively). Then, the best fit between the weighted sum of these convolutions and the time series at each voxel was computed by standard general linear modelling (GLM) and an estimate of the response (*β*) obtained for each experimental condition. As reported in our previous publications (Blasi et al., 2011, 2015), instead of the standard adult HRF, we adopted a strategy of obtaining HRF information directly from the data, whilst minimizing the statistical bias that could result from this approach. Reasoning that an auditory experiment should produce a dominant auditory response, we obtained the HRF in the auditory cortex for each subject by deconvolution from the mean time-series response in this brain region. For each infant, we then used the mean HRF estimated from all the other infants, thus producing the best estimate of the HRF unbiased by the infant being analysed. The data for each infant were then analysed using standard GLM analysis and the estimated unbiased HRF. This was repeated for all infants. In the last step of the data analysis, the data were normalized to Talairach space using an infant template, as previously described by our group (Blasi et al., 2011, 2015) and others (Dehaene-Lambertz, Dehaene, & Hertz-Pannier, 2002).

After normalization, a one-sample *t* statistic was computed at each voxel using the *β* estimates for each individual. The significance of this *t* statistic was then tested at a voxel-wise level by data permutation. Briefly, the signs of the *β*s were randomly permuted, and the resulting permuted *β*s used to recalculate a one-sample *t* statistic. Repeating this procedure 40 000 times per voxel produces the distribution of *t* under the null hypothesis without the requirement for the data to follow a normal distribution – an assumption often violated in small group fMRI data. The significance of the *t* obtained from the unpermuted data was assessed by reference to the probability distribution of *t* obtained by data permutation. We used an uncorrected *p* value of 0.005 for voxel-wise maps and we report activation in clusters as small as 3 voxels.

Based on the pattern of neural processing anomalies typically associated with groups at increased risk for depression, and to maximise the probability of detecting between-group differences, we contrasted emotionally positive (laughter) *v.* baseline, and emotionally negative (crying) sounds *v.* baseline in both groups. We then analysed the interaction between this contrast and infant exposure to PMD. To examine the underlying cause of between-group differences, we created interaction and regression plots.

Some previous studies report sex-specific programming effects following prenatal exposure to depression (Charil, Laplante, Vaillancourt, & King, 2010). Hence, we completed a *post hoc* group-by-sex two-way ANOVA.

Our study was underpowered to examine the effects of maternal prenatal SSRI use. However, we completed a *post hoc* within-group analysis of infants exposed to PMD by carrying out a 2-groups (SSRI+, SSRI–) × 2-sounds (emotion+, emotion–) ANOVA.

## Results

### Clinical characteristics

In the PMD exposed group, 29 infants were included in the current analyses. Eight infants were excluded as they woke up before the start (*n* = 2) or woke up or moved excessively during (*n* = 6) the fMRI task. In the non-PMD exposed group, 29 infants were included in the current analyses, with 12 excluded as they woke up before (*n* = 1) or woke up or moved excessively during (*n* = 6) the fMRI task. There were no significant differences in the clinical characteristics of the infants, or mothers, excluded. There were no statistically significant between-group differences with respect to *infant* age or birth weight at MRI, gestational age at birth, gender, or *maternal* age, educational level, breastfeeding or marital status (Table 1).

### FMRI imaging data

There were no significant group differences in motion measurements of translation or rotation of images.

**Fig. 1.**
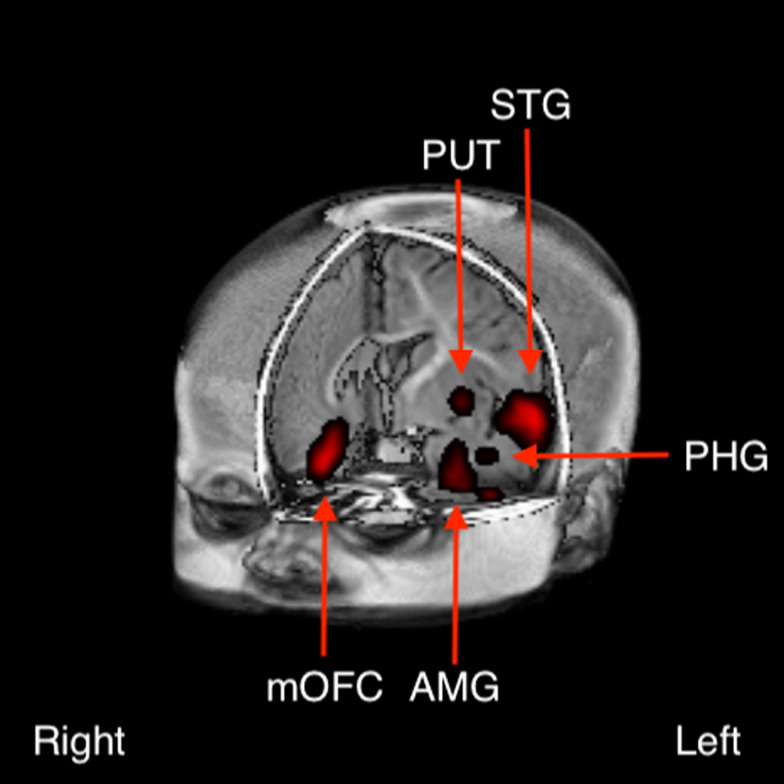
Between-group difference in brain activation to the contrast of emotional positive (laughter) *v*. negative (crying) sounds between infants born to depressed *v*. non-depressed mothers (*p* < 0.005). These included left dominant activations in the anterior temporal pole [predominantly in the superior temporal gyrus (STG)], amygdala (AMB), parahippocampal gyrus (PHG) and putamen (PUT), and right dominant activations in the medial orbitofrontal cortex (mOFC).

Following FDR correction for multiple comparisons (Benjamini & Yekutieli, 2001), between-group differences were detected in several clusters at *p* < 0.005 (Fig. 1). These included left dominant activations in the anterior temporal pole [predominantly superior temporal gyrus (STG)], striatum, amygdala/parahippocampal gyrus and putamen, and right dominant activations in the orbitofrontal cortex, and inferior frontal gyrus (IFG).

**Fig. 2.**
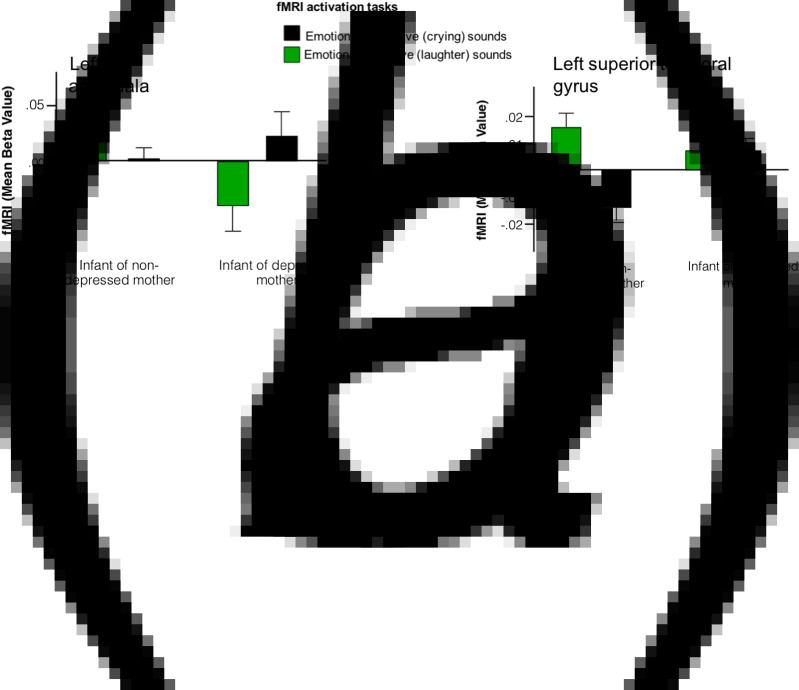
Interaction plots suggest that infants born to non-depressed mothers exhibit hyper-activation in response to emotionally positive sounds, whereas infants born to depressed mothers exhibit hyper-activation in response to emotionally negative sounds in regions including (*a*) left amygdala and (*b*) left superior temporal gyrus.

Interaction plots permitted statistical disentanglement of the conditions [i.e. positive (laughter) or negative (crying) valence sounds] in the clusters where the group differences were arising. We found a greater activation to emotionally positive contrasted with emotionally negative sounds in infants unexposed to PMD. Conversely, exposure to PMD was associated with greater (or unchanged) activation to emotionally negative contrasted with emotionally positive sounds in the clusters identified (Fig. 2).

### Sex differences

A *post hoc* group-by-sex two-way ANOVA did not find statistically significant sex differences in those clusters where the main effects of exposure to PMD depression were found.

### SSRI effects

With imagewise expectation of the number of false-positive clusters under the null hypothesis set at <1, the analysis did not find any difference between the two groups. Therefore, in our underpowered analysis of prenatal SSRI use, we did not detect any statistically significant differences or trends between the mothers with PMD with or without SSRI use.

## Conclusion

Our findings indicate that infants exposed to PMD exhibit differences in activation within a frontal-limbic neural network. More specifically, we report, for the first time, that infants born to mothers with PMD exhibit greater activation in response to emotionally negative sounds (crying), whereas infants unexposed to PMD demonstrate greater activation in response to emotionally positive sounds (laughter) (Fig. 1*a–c*). The finding of a differential response to positive and negative valanced sounds by 3–6 months old may have significant implications for our understanding of neural mechanisms that underpin the increased risk for later-life depression in this population. Whilst it was beyond the scope of the current study to assess this risk, we aim to follow-up our cohort longitudinally for later behavioural measures of depression.

The direction of findings within subcortical structures was consistent with our *a priori* hypothesis, and studies in people with MDD (Beesdo et al., 2009; Fu et al., 2004; Siegle et al., 2007; Surguladze et al., 2005; Zhong et al., 2016), and older children born to mothers with PMD (van der Knaap et al., 2017). However, the direction of activation in the prefrontal cortex (PFC; i.e. orbitofrontal cortex, and IFG) was opposite to our *a priori* hypothesis. In older subjects with depression, increased amygdala activation to negatively valanced stimuli is associated with reduced PFC activation. This is understood to be driven by *inhibitory* connections between the amygdala and PFC. It has previously been reported that these inhibitory connections do not fully develop until adolescence (Silvers et al., 2017), which could partly account for the synchronous activity in these regions reported in the infants studied. Further, a recent resting-state fMRI study, using dynamic causal modelling, has reported a stronger *excitatory* influence from the amygdala to the PFC, and a weaker excitatory influence from the PFC to the amygdala, in PMD-exposed infants compared with controls (Cha et al., 2017). We, and others, have also reported a positive correlation between prenatal stress and fractional anisotropy of the white matter tract linking these regions (i.e. uncinate fasciculus) in neonates (Rifkin-Graboi et al., 2015) and children (Sarkar et al., 2014). Importantly, a recent resting-state fMRI study, in 6-month-old infants exposed to PMD symptoms, has found that aberrant connectivity between the amygdala and PFC was independent of *postnatal* depression scores (Qiu et al., 2015). These findings lend further support to a growing consensus that the prenatal environment plays a critical role in the development of neural networks involved in emotion processing. While the *task-based* fMRI approach used in our study permitted us to take the next step and explore the interaction between this network and emotion-processing, interpretation of our findings remains unclear. In particular, it is unclear whether emotions construed by adults as happy (e.g. laughter) or sad (e.g. crying) are perceived similarly by 3–6 months old infants.

The mechanism(s) underpinning the relationship between PMD and the developing foetal brain remains unclear. However, research over the past decade is converging on a psycho-neuro-immunological model of ‘foetal programming’, with the maternal hypothalamic–pituitary–adrenal (HPA) axis modulating the relationship between maternal depression and the immune system [see Hantsoo, Kornfield, Anguera, & Epperson (2019) for review]. For example, we have previously reported that during pregnancy, PMD is associated with increased maternal HPA axis activity and maternal inflammatory cytokines (i.e. IL-6, IL-10, VEGF and TNF*α*) (Osborne et al., 2018). Further, at 12 months of age, both measures correlate positively with the infant cortisol response after a stressful event (i.e. immunisation). These findings support the hypothesis that placental transfer of maternal inflammatory cytokines and glucocorticoids programmes foetal cortisol stress reactivity. Further, a recent study in 12-month-old infants has found increased maternal prenatal IL-6 is associated with poorer cognitive function and reduced white matter integrity in a frontal-limbic network that incorporates the same regions modulated in our study (Rasmussen et al., 2019). Whilst these studies support a psychoneuroimmunological process underpinning the frontal-limbic aberrations found in our study, they have difficulty explaining the differential reaction to emotional sounds reported.

A mechanism that could partly explain *why* infants exposed to PMD react to emotional sounds differently is the ‘mismatch response’ (MMR). This is a characteristic neural response to the detection of unpredicted stimuli, and typically involves activation of the PFC (/IFG) and STG (Doeller et al., 2003). For example, presentation of pseudo-word sounds to foetuses *in utero* is associated with an enhanced MMR to re-exposure of the pseudo-word sounds when presented with a different (unpredicted) pitch postnatally (Partanen et al., 2013). Further, with particular relevance to our study, the MMR has been found to be elicited automatically, even during natural sleep (Cheour et al., 2002). Therefore, for example, increased exposure to maternal crying sounds *in utero* would predict an enhanced MMR to differences in the pitch/tone of crying sounds postnatally. This could neatly account for our group differences in PFC and STG. However, whilst some studies report a positive correlation between depression and crying (Vingerhoets, Rottenberg, Cevaal, & Nelson, 2007), prenatal exposure to specific sounds (e.g. maternal crying) was not measured and this needs to be tested in future research.

Finally, whilst our study has a number of strengths, there are a number of additional factors that also need to be addressed in future studies. One important issue is the potential role of prenatal SSRI exposure on offspring born to depressed mothers. Whilst we did not find any significant differences in brain activation associated with SSRIs in the depressed group, our study was underpowered to fully test this. Further, it would be beneficial for future studies to fractionate infants based on their genotype as, for example, different allelic variants of the of 5-HTT promoter have been reported to modulate the effects of SSRIs differently (Serretti, Kato, De Ronchi, & Kinoshita, 2007). Also, studies would also benefit from including measures of prenatal anxiety, personality disorders and postnatal depression. Research suggests, for example, that prenatal depression is a predictor of postnatal depression (Milgrom et al., 2008) and exposure to either could modulate the developing infant brain. Whilst the assumption of the current study is that the effects reported are mediated by exposure to prenatal depression, the role of maternal depression during the 3–6 months prior to infant scanning cannot be excluded. Further, subsequent studies would benefit from recruiting mothers with a positive history of depression, but no prenatal depression. The addition of this group would assist in separating out the relative roles of state *v.* trait factors (Hannigan et al., 2018).

It is anticipated that replication of our findings, powered to analyse the influence of SSRIs and genetic factors, will lead to a greater understanding of the effects of PMD and medication on neurodevelopment. Further, it is hoped that these findings will permit more accurate prenatal counselling to depressed women about the putative risks of untreated prenatal depression on infant brain, and towards better treatments to reduce the risk for psychiatric morbidity in exposed offspring in later life.

## Data

Authors had access to the study data and access is on-going. Data associated with a manuscript are available on written request.
